# Development of an *in-vitro* model system to investigate the mechanism of muscle protein catabolism induced by proteolysis-inducing factor

**DOI:** 10.1038/sj.bjc.6600236

**Published:** 2002-05-03

**Authors:** M C C Gomes-Marcondes, H J Smith, J C Cooper, M J Tisdale

**Affiliations:** Department of Physiology and Biophysics, University of Campinas, UNICAMP, SP, Brazil 13083-970; Pharmaceutical Sciences Research Institute, Aston University, Birmingham B4 7ET, UK

**Keywords:** cachexia, proteolysis-inducing factor (PIF), myotubes, ubiquitin-proteasome proteolysis

## Abstract

The mechanism of muscle protein catabolism induced by proteolysis-inducing factor, produced by cachexia-inducing murine and human tumours has been studied *in vitro* using C_2_C_12_ myoblasts and myotubes. In both myoblasts and myotubes protein degradation was enhanced by proteolysis-inducing factor after 24 h incubation. In myoblasts this followed a bell-shaped dose-response curve with maximal effects at a proteolysis-inducing factor concentration between 2 and 4 nM, while in myotubes increased protein degradation was seen at all concentrations of proteolysis-inducing factor up to 10 nM, again with a maximum of 4 nM proteolysis-inducing factor. Protein degradation induced by proteolysis-inducing factor was completely attenuated in the presence of cycloheximide (1 μM), suggesting a requirement for new protein synthesis. In both myoblasts and myotubes protein degradation was accompanied by an increased expression of the α-type subunits of the 20S proteasome as well as functional activity of the proteasome, as determined by the ‘chymotrypsin-like’ enzyme activity. There was also an increased expression of the 19S regulatory complex as well as the ubiquitin-conjugating enzyme (E2_14k_), and in myotubes a decrease in myosin expression was seen with increasing concentrations of proteolysis-inducing factor. These results show that proteolysis-inducing factor co-ordinately upregulates both ubiquitin conjugation and proteasome activity in both myoblasts and myotubes and may play an important role in the muscle wasting seen in cancer cachexia.

*British Journal of Cancer* (2002) **86**, 1628–1633. DOI: 10.1038/sj/bjc/6600236 www.bjcancer.com

© 2002 Cancer Research UK

## 

Progressive loss of lean body mass is the most debilitating and life-threatening aspect of cancer cachexia. There may be as much as 75% loss of skeletal muscle mass while the non-muscle protein compartment is preserved (Fearon, 1992), thus distinguishing this syndrome from that of simple starvation. Muscle mass is determined by both the rate of protein synthesis and the rate of protein degradation, and alteration in the balance between these two events can lead to hypertrophy or atrophy of muscle. Thus a reduced rate of protein synthesis and an increased rate of protein degradation was found in newly diagnosed cancer patients with weight loss (Lundholm *et al*, 1976). In this study an increase in cathepsin D activity was found in biopsies from the rectus abdominal muscle and this correlated with the increased rate of protein degradation.

Skeletal muscle contains multiple proteolytic systems for the breakdown of proteins including lysosomal, calcium-dependent and ATP-ubiquitin-dependent proteolytic pathways (Lecker *et al*, 1999). However, skeletal muscle contains few lysosomes and cathepsins are not involved in the breakdown of myofibrillar proteins (Lowell *et al*, 1986). In addition mRNA for cathepsin B or cathepsin B and B + L activities were not found to change in skeletal muscle of rats implanted with a cachexia-inducing tumour (Temparis *et al*, 1994), although in the MAC16-induced cachexia in mice there were increases in both cathepsins B and L (Lorite *et al*, 1998). However, the contribution of this pathway and the calcium-dependent proteolytic process to overall protein loss is small and the ATP-ubiquitin-dependent proteolytic pathway is generally considered to be mainly responsible for muscle protein catabolism in cancer cachexia.

We have been investigating the role of a sulphated glycoprotein, produced exclusively by cachexia-inducing tumours in both mouse and man, in the catabolism of skeletal muscle in cancer cachexia (Todorov *et al*, 1996a). Urinary excretion of the glycoprotein could be detected in patients with carcinomas of the pancreas, liver, rectum, colon, breast, lung and ovary, where the weight loss was greater than or equal to 1 kg per month (Cariuk *et al*, 1997). This material has been named proteolysis-inducing factor (PIF) because of the ability to induce muscle protein catabolism directly, both in isolated skeletal muscle (Lorite *et al*, 1997) and in murine myoblasts *in vitro* (Smith *et al*, 1999). Administration of PIF to non-tumour bearing mice produced a rapid decrease in body weight (8.6% in 24 h), with specific loss of lean body mass (Lorite *et al*, 1998). In PIF-treated animals there was loss of skeletal muscle, while visceral protein in heart and kidney was conserved, and there was even an increase in weight of the liver. Using inhibitors of the proteolytic pathways, protein catabolism in skeletal muscle was found to be mediated entirely through an ATP-dependent pathway (Lorite *et al*, 1998).

Further studies in mice (Lorite *et al*, 2001) have shown PIF to increase both mRNA and protein levels of ubiquitin, the *M*_r_ 14 000 ubiquitin carrier protein, E2_14k_, and proteasome subunits in gastrocnemius muscle, but not in heart, suggesting activation of the ATP-ubiquitin-dependent proteolytic pathway. A similar effect was seen in murine myoblasts. However, it is possible that *in vivo* PIF could stimulate expression of other factors, which may be responsible for the effect, while myoblasts are not the best model to study protein degradation in muscle, since they do not contain the myofibrillar proteins actin and myosin.

In order to develop an *in vitro* system for further mechanistic studies a comparison has been made of the effects of PIF on both murine myoblasts and myotubes, which contain myofibrillar proteins. This study examines the effect of both PIF concentration and exposure time on the activity of the ubiquitin proteasome proteolytic pathway using immunoblotting to determine the expression of E2_14k_, 20S and 19S proteasome subunits, while the functional activity of the proteasome has been determined by measuring the ‘chymotrypsin-like’ enzyme activity, the major proteolytic activity of the proteasome, as well as myosin expression in myotubes.

## MATERIALS AND METHODS

### Materials

L-[2,6-^3^H] Phenylalanine (sp.act. 2.00 TBq mmol^−1^) was purchased from Amersham International (Bucks, UK). Foetal calf serum (FCS), horse serum (HS) and Dulbecco's modified Eagle's medium (DMEM) were purchased from Life Technologies (Paisley, UK). Mouse monoclonal antibody to 20S proteasome subunits α1, 2, 3, 5, 6 and 7 (clone MCP 231) was purchased from Affiniti Research Products, Exeter, UK, and mouse monoclonal antibody to myosin heavy chain was from Novocastra, Newcastle, UK. Rabbit polyclonal antisera to ubiquitin conjugating enzyme (E2_14k_) was a gift from Dr Simon Wing, McGill University, Montreal, Canada and rabbit polyclonal antisera to the 20S proteasome β-subunit was from Calbiochem, Nottingham, UK. Mouse anti-MSSI and anti-p42 antibody were a gift from Dr Jane Arnold, UK.

### Cell culture

The C_2_C_12_ mouse myoblast cell line was cultured in DMEM supplemented with 12% FCS, 1% nonessential amino acids, and 1% penicillin-streptomycin, in a humidified atmosphere of 5% CO_2_ in air at 37°C. Experiments were performed on cells in the subconfluent state. Myotubes were formed by allowing confluent cultures to differentiate for 9 days in DMEM containing 2% HS with changes of medium every 2 days.

### Purification of PIF

PIF was purified from solid MAC16 tumours from mice with weight loss between 20 and 25%. The tumour homogenate was precipitated with ammonium sulphate (40% w v^−1^), and the supernatant subjected to affinity chromatography using a monoclonal antibody immobilized to a protein A matrix, as described (Todorov *et al*, 1996a). The immunogenic fractions were concentrated and used for studies without further purification, since the major contaminant was albumin (Todorov *et al*, 1996b). There was no detectable endotoxin in purified PIF preparations.

### Measurement of total protein degradation

C_2_C_12_ myoblasts were seeded at 4×10^4^ cells per well in 2 ml DMEM in 6-well multidishes. After 24 h cells were labelled with L-[2,6-^3^H] phenylalanine (0.67 mCi mmole^−1^) for a further 24 h period, washed three-times in PBS and incubated in fresh DMEM without phenol red (2 ml) in the presence of various concentrations of PIF for a further 24 h period. The amount of radioactivity released into the medium was determined using a 2000CA Tri-Carb liquid scintillation analyzer. Protein degradation in myotubes was determined by the same method.

### Measurement of proteasome activity

The chymotrypsin-like activity of the proteasome was determined fluorimetrically according to the method of Orino *et al* (1991), with some modifications. Cells were washed twice with ice-cold PBS and scraped from the substratum into 20 mM Tris HCl, pH 7.5, 2 mM ATP, 5 mM MgCl_2_ and 1 mM dithiothreitol (0.5 ml). The cells were dissociated by sonication with three pulses of 15 s with 10 s intervals at 4°C. The sonicate was then centrifuged for 10 min at 15 000 r.p.m. at 4°C and the resulting supernatant (0.1 ml) was used to determine chymotrypsin-like activity using the fluorogenic substrate succinyl-LLVY-MCA (0.1 mM) in a total volume of 0.2 ml of 100 mM Tris HCl, pH 8.0, with or without 10 μM lactacystin for 1 h on ice. The reaction was terminated by the addition of 80 mM sodium acetate, pH 4.3 (1 ml) and the fluorescence determined with an excitation wavelength of 360 nm and an emission wavelength of 460 nm, after further dilution with 2 ml 80 mM sodium acetate. The activity was adjusted for the protein concentration of the sample, determined using the Bradford assay (Sigma Chemical Co., Dorset, UK).

### Western blot analysis

Samples of cytosolic protein (2.5 to 5 μg) were resolved on 12% sodium dodecylsulphate, polyacrylamide gels (SDS–PAGE) and transferred to 0.45 μm nitrocellulose membranes (Hybond A, Amersham, UK), which had been blocked with 5% Marvel in Tris buffered saline, pH 7.5, at 4°C overnight. The primary antibodies were used at a 1 : 1500 dilution, except for the 20S proteasome β subunit used at a 1 : 2000 dilution and myosin heavy chain, used at 1 : 250 dilution. The secondary antibodies were peroxidase conjugated, either goat anti-rabbit (Sigma Chemical Co., Dorset, UK) or rabbit anti-mouse (Amersham, UK) and were used at a 1 : 1500 dilution. Incubation was for 1 h at room temperature and development was by enhanced chemiluminescence (ECL) (Amersham, UK). Blots were scanned by a densitometer to quantitate differences, and a parallel gel was silver stained to ensure equal loading. The densitometry results were analysed using ‘Phoretix ID Advanced’ software.

### Statistical analysis

Results were expressed as mean±s.e.m. Differences were determined by one-way ANOVA.

## RESULTS

**Figure 1:**
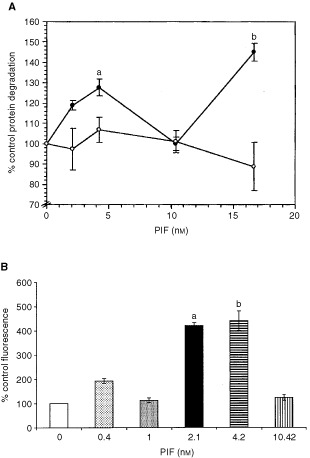
(**A**) Effect of PIF on total protein degradation in C_2_C_12_ myoblasts, either in the presence of excess phenylalanine (2 mM) (solid circle) or cycloheximide (1 μM) (open circle). Measurements were made 24 h after the addition of PIF and are shown as mean±s.e.m where *n*=6. Differences from controls in the absence of PIF are indicated as a, *P*<0.05 or b, *P*<0.01. (**B**) Chymotryptic activity of soluble extracts of C_2_C_12_ myoblasts determined using the fluorogenic substrate sucLLVY-MCA after treatment with PIF for 24 h. Results are shown as mean±s.e.m where *n*=9.

The effect of PIF on protein degradation in C_2_C_12_ myoblasts after 24 h incubation, as determined by the release of L-[2,6-^3^H]phenylalanine into the culture medium, in the presence of excess (2 mM)phenylalanine is shown in Figure 1A

. PIF enhanced protein degradation maximally at concentrations between 2.1 and 4.2 nM, with some indication of an enhanced degradation also at 16.7 nM PIF. Protein degradation was not enhanced by PIF in the presence of cycloheximide (1 μM), showing that PIF stimulation of protein degradation required new protein synthesis.

**Figure 2:**
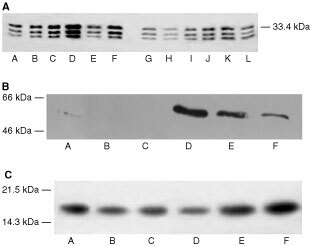
(**A**) Western blot of soluble extracts of C_2_C_12_ myoblasts detected with MCP231, a murine monoclonal antibody to the proteasome α-subunits after treatment of cells with PBS (A) or 1 (B), 2 (C), 4 (D), 10.5 (E) or 17 nM PIF (F) alone or with cycloheximide (1 μM) (G) or with cycloheximide (1 μM) plus 1 (H), 2 (I), 4 (J), 10.5 (K) or 17 nM PIF (L). (**B**) Western blot analysis of soluble extracts of C_2_C_12_ myoblasts either untreated (A) or treated with 0.4 (B), 1 (C), 2 (D), 4 (E) or 10 nM PIF (F) and detected with mouse anti-human MSSI antibody. (**C**) Western blot analysis of soluble extracts of C_2_C_12_ myoblasts either untreated (A) or treated with 0.4 (B), 1 (C), 2 (D), 4 (E) or 10 nM PIF (F) and detected with E2_14k_ rabbit polyclonal antisera.

**Figure 3:**
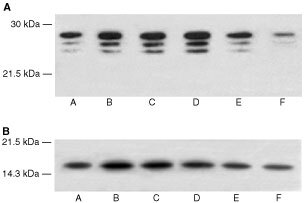
Western blot of soluble extracts of C_2_C_12_ myoblasts treated with PIF for 3 h and detected with MCP231 antibody (**A**) or E2_14k_ rabbit polyclonal antisera (**B**).

To determine whether PIF-induced protein catabolism was mediated through the ATP-ubiquitin-dependent pathway proteasome functional activity was determined by measuring ‘chymotrypsin-like’ enzyme activity, the major proteolytic activity of the β-subunits. Using the fluorogenic substrate succinyl LLVY-MCA an increase in enzyme activity was detectable at concentrations of PIF between 2 and 4 nM (Figure 1B). The effect on protein expression of proteasome subunits in the presence of PIF was determined by immunoblotting. Cellular supernatants of PIF-treated cells were Western blotted using MCP 231 antibody, a murine monoclonal to the 20S proteasome, which reacts with the six different α-type subunits. Three bands were detected at approximate molecular weight of 29 000, 32 000 and 35 000 (Figure 2A

). There was an increase in expression of all three α-subunits in the presence of PIF, with a dose-response curve similar to that for protein degradation (Figure 1A), and with maximal induction at 4.2 nM PIF. This effect was also attenuated in the presence of cycloheximide. The effect of 24 h incubation with PIF on the expression of the ATPase subunit, MSSI, of the 19S proteasome regulatory complex is shown in Figure 2B. A single band of *M*_r_ ∼ 50 000 was apparent, which was consistent with the predicted amino acid sequence of the ATPase MS73 (Dawson *et al*, 1995). As with proteasome α-subunits maximal expression was seen at concentrations of PIF between 2 and 4 nM (500 and 300% of the control respectively). The effect of PIF on the expression of the *M*_r_ 14 000 ubiquitin-conjugating enzyme (E2_14k_) is shown in Figure 2C. The antibody recognises both isoforms of E2_14k_, including the isoform for which mRNA levels increase in atrophying muscle, as a single band at *M*_r_ 17 000 (Rajapurohitam *et al*, 1999). PIF caused an increase in expression of E2_14k_ in a concentration-dependent manner, with maximal expression at 4.2 and 10 nM (125 and 183% of control respectively). There was evidence for an increase in expression for both proteasome α-subunits (Figure 3A

) and E2_14k_ (Figure 3B) within only 3 h of PIF incubation, although at this time point lower concentrations of PIF (0.4 and 1 nM) were effective. This provides some evidence for co-ordinate upregulation of ubiquitin conjugating enzymes and proteasome subunits.

**Figure 4:**
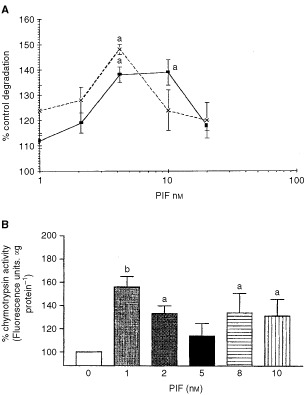
(**A**) Effect of PIF on total protein degradation in C_2_C_12_ myotubes after 24 h (×) and 48 h (solid square) incubation. Measurements were made in the presence of excess (2 mM) phenylalanine and are the average of nine determinations. Differences from controls in the absence of PIF are indicated as a, *P*<0.05 and b, *P*<0.01. (**B**) Chymotryptic activity of soluble extracts of C_2_C_12_ myotubes after treatment with PIF for 24 h. Values shown represent mean±s.e.m where *n*=8. Differences from controls in the absence of PIF are shown as a, *P*<0.05 and b, *P*<0.01.

Further studies were carried out using C_2_C_12_ myotubes, since these contain the myofibrillar proteins actin and myosin, characteristic of skeletal muscle. The effect of PIF on total protein catabolism, as determined by [^3^H]phenylalanine release is shown in Figure 4A

**Figure 5:**
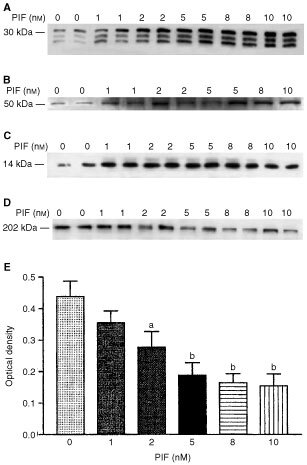
Western blot of soluble extracts of C_2_C_12_ myotubes either untreated or treated with PIF at a concentration of 1, 2, 5, 8 or 10 nM for 24 h and detected with either (**A**) MCP231 antibody directed to the proteasome α-subunits, (**B**) Mouse anti-human MSSI antibody, (**C**) E2_14k_ rabbit polyclonal antiserum, or (**D**) Mouse monoclonal antibody directed to myosin heavy chain. (**E**) Shows a densitometric analysis of myosin heavy chains from four separate blots, *n*=8. Differences from control are shown as a, *P*<0.05 and b, *P*<0.01.

**Figure 6:**
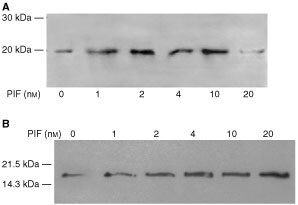
Western blot of soluble extracts of C_2_C_12_ myotubes either untreated or treated with PIF at a concentration of 1, 2, 4, 10 or 20 nM for 48 h and detected with either 20S proteasome β-subunit rabbit polyclonal antisera (**A**) or E2_14k_ rabbit polyclonal antisera (**B**).

. Like the myoblasts there was an increased protein breakdown in the presence of PIF with a maximal effect at 4.2 nM at 24 h, and the effect was still apparent at 4.2 and 10 nM PIF after 48 h without further addition of PIF to the culture system. The effect of PIF on proteasome proteolytic activity after 24 h, as determined by the ‘chymotrypsin-like’ enzyme activity is shown in Figure 4B. A significant increase in enzyme activity was seen at all concentrations of PIF between 1 and 10 nM. There was also an increased expression of the 20S proteasome α-subunits at all concentrations of PIF between 1 and 10 nM, which increased with increasing concentrations of PIF, with a 2.6-fold enhancement at 10 nM PIF (*P*<0.01 from control by densitometric analysis) (Figure 5A

). Expression of MSSI was also enhanced at all concentrations of PIF ranging between 4.6-fold at 1 nM (*P*<0.01 from control) to 7.2-fold at 10 nM PIF (*P*<0.05 from control) (Figure 5B). PIF-treatment also induced an increase in expression of p42, an ATPase subunit of the 19S regulator that promotes ATP-dependent association of the 20S proteasome with the 19S regulator to form the 26S proteasome (Tanahashi *et al*, 1999), but there was less of a clear-cut dose–response relationship. A similar increase in expression was seen with E2_14k_ (Figure 5C). All concentrations of PIF above 1 nM produced a significant (*P*<0.01 from control) increase in E2_14k_, which with a 2.8-fold enhancement at 10 nM was similar to that observed with the 20S α-subunit expression (Figure 5A). Myosin expression was reduced as the concentration of PIF increased (Figure 5D, E) in inverse proportion to activation of expression of the 20S proteasome (Figure 5A). There was still an increased expression of the proteasome, as detected by the β-subunit, 48 h after the original addition of PIF at concentrations of PIF between 10 nM (three-fold) and 1 nM (two-fold) (Figure 6A

). There was also an increased expression of E2_14k_ (1.5-fold) at these concentrations, but also an increased expression (two-fold) at 20 nM PIF (Figure 6B). Since protein degradation was not increased at 20 nM PIF (Figure 4A) this suggests that proteasome subunit induction rather than ubiquitin-conjugating enzymes are rate-limiting in protein catabolism.

## DISCUSSION

The ubiquitin-proteasome system is the major pathway for selective protein degradation in eukaryotic cells, and is involved not only in protein catabolism in skeletal muscle, but numerous other processes, such as progression of the cell cycle, transcription, development, growth, and atrophy of developed tissues (Kornitzer and Ciechanover, 2000). In this process proteins are marked for degradation by attachment of a polyubiquitin chain, via the ubiquitin activating enzyme (E1), which conjugates ubiquitin through a thioester bond in an ATP-requiring reaction; a trans-esterification reaction, whereby the activated ubiquitin is transferred to a cysteine residue in the active site of the ubiquitin-conjugating enzyme (E2), suggested to be a rate-limiting step in the pathway (Wing and Banville, 1994), and a ubiquitin-protein ligase (E3), which is responsible for substrate recognition and polyubiquitination. Polyubiquitinated proteins are degraded into small peptides by a large multicatalytic protease, the 26S proteasome. This pathway plays an important role in muscle protein degradation induced, not only in cancer cachexia (Temparis *et al*, 1994; Lorite *et al*, 1998), but also in starvation (Wing and Goldberg, 1993), sepsis (Tiao *et al*, 1994), metabolic acidosis (Mitch *et al*, 1994) and denervation atrophy (Medina *et al*, 1995).

Previous studies (Smith *et al*, 1999) have established the C_2_C_12_ murine myoblast cell line to be an appropriate surrogate model for studying protein degradation induced by PIF, possibly through the mediation of 15-HETE. In this study we have shown PIF to co-ordinately upregulate the proteasome chymotrypsin-like activity in this cell line, as well as expression of the 20S proteasome α-type subunits, MSSI, an ATPase subunit of the 19S complex and the *M*_r_ 14 000 E2 ubiquitin-conjugating enzyme, as determined by Western blotting. We have chosen to measure the chymotrypsin-like activity of the proteasome in view of recent observations that this is rate limiting in proteasome-dependent protein degradation (Kisselev *et al*, 1999). Although most studies have measured mRNA as a measure of gene expression we have measured intracellular protein levels and functional activity of the proteasome, because of reports (Kanayama *et al*, 1991; Shimbara *et al*, 1992) that in various cells elevated concentrations of mRNA of proteasome subunits were not found to be accompanied by increased concentrations or activities of proteasomes. In addition we have chosen to measure E2_14k_ and 20S/19S proteasome expression as a measure of the ubiquitin-proteasome pathway, rather than changes in ubiquitin expression and conjugation, because an increase in ubiquitin may signal an increased cell death through apoptosis (Soldatenkov and Dritschilo, 1997), rather than an increase in proteolysis. Thus, although the enhanced protein degradation in rat skeletal muscle *in vivo* after TNF-α administration is associated with an increase in gene transcription, and higher levels of free and conjugated ubiquitin (Garcia-Martinez *et al*, 1993), there is no evidence for an increase in proteasome expression *in vitro* (Ebisui *et al*, 1995), although an increased ubiquitin gene expression was still observed (Llovera *et al*, 1997). In the muscles of mice bearing the colon 26 tumour loss of muscle mass was associated with an increase in expression of both polyubiquitin and proteasome subunits. Treatment with an anti-IL-6 receptor antibody reduced polyubiquitin expression, but had no effect on expression of proteasome subunits (Tsujinaka *et al*, 1996).

As in a previous study (Smith *et al*, 1999) PIF-induced protein degradation in C_2_C_12_ myoblasts followed a bell-shaped dose-response curve with maximal effects at a PIF concentration between 2 and 4 nM. In contrast there was no evidence of down-regulation of protein degradation in myotubes with an increased level found above a threshold concentration of 1 nM PIF. However, the concentration of PIF required to induce protein catabolism was similar in both myoblasts and myotubes. Protein degradation induced by PIF in myoblasts was completely attenuated in the presence of cycloheximide, suggesting the requirement for new protein synthesis to affect protein catabolism. In both myoblasts and myotubes PIF induced co-ordinate upregulation of both 20S proteasome α-subunits and the 19S regulator, as well as E2_14k_. Proteasome functional activity, as determined by the ‘chymotrypsin-like’ enzyme activity, was also increased in both cell lines at concentrations of PIF enhancing expression of the proteasome α-subunits, while in myotubes the expression of myosin was decreased.

This study shows co-ordinate upregulation of both the 20S proteasome α-subunits and the 19S regulator, MSSI, an ATPase, which is thought to provide energy, both for the association of the 20S proteasome with the 19S complex, and to inject the substrate into the proteolytic chamber of the 20S proteasome. MSSI was found to be increased in the wasting muscle of rats bearing the Yoshida sarcoma (Attaix *et al*, 1997), and has been suggested to facilitate the rapid proteolysis of muscle proteins in cancer cachexia. Expression of MSSI has been suggested to be regulated independently of the 20S proteasome subunits, since the increased expression of MSSI was normalised in cachectic rats given pentoxifylline, but not tobafylline, although both suppressed the enhanced proteolysis (Attaix *et al*, 1997). However, in the present study the expression of both MSSI and the 20S proteasome α-subunits appeared to be regulated co-ordinately, suggesting central control of the whole cascade by PIF. The effect of PIF was evident within as little as 3 h and persisted for a 48 h period. However, at 48 h E2_14k_ expression was elevated at concentrations of PIF that had no effect on protein degradation. This suggests that ubiquitin-conjugation is not rate-limiting for proteolysis and that this requires an induced expression of proteasome subunits. This conclusion is similar to that reached by Temparis *et al* (1994), from a study in rats bearing the Yoshida sarcoma, where a significant reduction in protein mass was observed in the extensor digitorum longus (EDL) muscle close to the tumour, but not in the tibalis anterior (TA) muscle. An increased mRNA expression for ubiquitin, E2_14k_ and the proteasome subunits C8 and C9 was found in the EDL muscle, but not in the TA muscle, where only mRNA for ubiquitin and E2_14k_ was increased. This suggested that increased mRNA expression for ubiquitin and E2_14k_ came first, but that increased gene expression of proteasome subunits was essential for enhanced protein catabolism. This conclusion is also supported from studies of skeletal muscle during cancer cachexia where an elevation of the high molecular mass conjugates of ubiquitin is observed (Lorite *et al*, 1998), suggesting that this step is not rate limiting in proteolysis. Thus proteasome catalytic activity rather than substrate ubiquitination appear to be the rate-limiting step in protein catabolism.

Further studies will concentrate on the mechanism by which PIF leads to upregulation of the ubiquitin-proteasome pathway and inhibitors of this process.
